# Martin Woesler, Hans-Martin Sass (Hrsg) (2020) Medizin und Ethik in Zeiten von Corona: LIT-Verlag, Berlin, Reihe: Ethik in der Praxis/Practical Ethics – Studien/Studies Bd. 48, 208 Seiten, 34,90 €/34,90 CHF, br., ISBN 978-3-643-14768-4

**DOI:** 10.1007/s00481-021-00632-3

**Published:** 2021-04-30

**Authors:** Anna-Henrikje Seidlein

**Affiliations:** Institut für Ethik und Geschichte der Medizin, Universitätsmedizin Greifswald, Ellernholzstr. 1–2, 17487 Greifswald, Deutschland

Das im Herbst 2020 erschienene Herausgeberwerk „Medizin und Ethik in Zeiten von Corona“ von Martin Woesler und Hans-Martin Sass reiht sich in den aktuellen Diskurs um die mit der COVID-19 Pandemie einhergehenden ethischen Fragen auf unterschiedlichen gesellschaftlichen Ebenen (Mikro‑, Meso- und Makroebene) ein. Zum Zeitpunkt des Abfassens der Rezension, rund ein Jahr nachdem die WHO die Sars-CoV‑2 Pandemie ausrief, hat die „Pandemiemüdigkeit“ in Deutschland den (oder „einen“?) Höhepunkt erreicht. Dementsprechend schwer wird es vermutlich einigen Leser:innen fallen, ein Buch zur Hand zu nehmen, das ihnen die aktuellen Probleme noch einmal schonungslos und aus anderen, teils neuen Perspektiven vor Augen führt.

Die potenzielle Leserschaft profitiert von dem zeitgleichen Erscheinen des Buches in deutscher und englischer Sprache; die Adressat:innen dürften gemäß der Reihe, in der das Werk erschienen ist, eine breite Zielgruppe aus Forschung und Praxis sein.

Der Sammelband zeigt in seinen 16 Beiträgen von Autor:innen aus fünf Ländern ein breites Spektrum der Problemlagen aus unterschiedlichen Disziplinen heraus (u. a. juristische, theologische und medizinische Perspektiven) auf, das sich aus der COVID-19 Pandemie ergibt. Entsprechend statuiert M. Woesler, dass „Dieser Band verwirklicht, was die Regierungen unserer Welt noch lernen müssen: In Zeiten der globalen Krise vereint zu sein“ (S. 2).

Das Buch gliedert sich in die fünf Abschnitte „Verteilungsfragen“, „Verantwortung“, „Ethische Probleme und Konsequenzen“, „Sozio-Ökonomie und die Arbeitswelt“ sowie „Theologische Reflexion und Ausblick“ und spiegelt damit die Vielfalt der (ethischen) Themen im Hinblick auf die – alle Lebensbereiche bestimmende – Pandemie wider.

Entsprechend der Dynamik einer Pandemie ist den Herausgebern darin zuzustimmen, dass die in ihrem Band vereinten Antworten auf die globale Krise schon „Bald […] historischen Wert haben“ (S. 1). Derzeit stellt sich die Situation jedoch noch so dar, dass die Beiträge nichts an ihrer Aktualität eingebüßt haben. Viele Problemfelder sind nach wie vor tagesaktuell in Entwicklung befindlich und die assoziierten Fragen ungeklärt (vgl. z. B. „Epistemische und ethische Probleme des ‚Risikogruppenbegriffs‘“ von A. Kruse) sowie zahlreiche daraus resultierende ethische und rechtliche Unsicherheiten bspw. im Zusammenhang mit der Ex-Post Triage (vgl. „Verteilen – zuteilen – vorenthalten in der Corona-Pandemie“ von J. Taupitz) bestehen fort. Einige der dargestellten Kontroversen gewinnen sogar mit der „dritten Welle“ gerade noch einmal an Bedeutung (vgl. „Immunitätsnachweise – vor der Einführung schon umstritten“ von K. Schlögl-Flierl). Die Herangehensweise, mit der die Autor:innen sich ihren Problemfeldern bzw. Fragestellungen nähern, sind methodisch vielfältig: unter anderem ethisch-theoretische Reflexionen, Systematisierung und Diskussion aktueller empirischer Befunde, international-vergleichende Analyse. So weisen einzelne Beiträge narrative Elemente auf – was sicher auch darin begründet ist, dass die Datenlage entsprechend der beleuchteten Phänomene unterschiedlich solide war bzw. immer noch ist. Ausgehend von einem persönlichen Erlebnis präsentiert z. B. D. Martin („Ethik in Zeiten von Corona“) seine Überlegungen zu den Besuchsregelungen in Krankenhäusern.

Dezidiert ethische Perspektiven kommen in einigen Beiträgen zu kurz bzw. werden nicht ausreichend expliziert. So stellt z. B. P. Melot de Beauregard in „Strategien für die neue Arbeitswelt“ die arbeitsrechtliche Perspektive in den Mittelpunkt; ethische Anknüpfungspunkte werden nicht aufgegriffen.

Hervorzuheben ist, dass viele Autor:innen sehr klar Position beziehen und persönliche Empfehlungen für anstehende Entscheidungen aussprechen sowie Forderungen an die entsprechenden Akteur:innen formulieren. Es liegt in der Natur der Sache, dass zu einigen Themen mehr Fragen aufgeworfen als Antworten geliefert werden können. Die je nach Blickwinkel entwickelten Fragen – auch sehr grundsätzlicher Natur – eröffnen jedoch interessante Perspektiven (vgl. v. a. „Wie kann Gott das zulassen“ von M. Eberle, „Die syndemische Perspektive und die Notwendigkeit einer gesundheitlichen Hermeneutik“ von F. Lolas).

Die bisweilen eigenwilligen sprachlichen Formulierungen, welche einer Übersetzung mancher Aufsätze aus der englischen Sprache geschuldet sein mögen, erschweren zeitweise den Lesefluss und die Möglichkeit, als Leser:in der Argumentation zu folgen (vgl. z. B. „Eine neue Bioethik“ von H. Hubenko).

Insgesamt eignet sich das Werk, um einen Einblick in das komplexe und vielseitige Feld der (nicht nur) ethischen Problemlagen zu gewinnen. Es sensibilisiert darüber hinaus für bislang im Diskurs wenig berücksichtigte Aspekte (vgl. „Individuelle Verantwortung und soziale Interaktion nach der Corona-Krise“ von C. Kaminsky). Leser:innen erhalten außerdem zugleich eine Vorstellung davon, welche Herausforderungen uns im Rahmen der Pandemiebewältigung noch bevorstehen, und zugleich Impulse für noch ausstehende Forschungs- und Innovations-Arbeit.

